# Supplementary material for: Stage-Specific Effects of Ionizing Radiation during Early Development
Source: Int J Mol Sci. 2020 Jun 1;21(11):3975. doi: 10.3390/ijms21113975 (PMC7312565; doi:10.3390/ijms21113975)
Supplement: Supplementary file 1 [file ijms-21-03975-s001.pdf]

Supplementary Table S1. The difference of cellular response to ionizing radiation among species. +: effective; −: defective; N.D.: Not Determined.

**Species**	**Cell Cycle Checkpoints**	**Predominant DNA Repair Pathway**	**Apoptosis**
Mammals	G1/S checkpoint: −<br>G2/M checkpoint: +	Homologous recombination	From two-cell stage in mouse
Xenopus	After midblastrula transition	N.D.	After onset of gastrulation
Fish	After midblastrula transition in zebrafish	N.D.	After mid-gastrulation stage in zebrafish
